# Evaluation of Cytotoxicity and Oxidative Stress of Whole Aerosol from Vuse Alto ENDS Products

**DOI:** 10.3390/toxics12020129

**Published:** 2024-02-04

**Authors:** Brian M. Keyser, Robert Leverette, John Wertman, Tom Shutsky, Reagan McRae, Ken Szeliga, Patrudu Makena, Kristen Jordan

**Affiliations:** RAI Services Company, Winston-Salem, NC 27106, USA; leverer@rjrt.com (R.L.); wertmaj1@rjrt.com (J.W.); szeligk@rjrt.com (K.S.); makenap@rjrt.com (P.M.); jordank2@rjrt.com (K.J.)

**Keywords:** whole aerosol, cigarette smoke, Vuse Alto ENDS, air–liquid interface, EpiAirway™, cytotoxicity, oxidative stress, Nrf2 luciferase reporter

## Abstract

Assessment of in vitro cytotoxicity is an important component of tobacco product toxicological evaluations. However, current methods of regulatory testing involve exposing monolayer cell cultures to various preparations of aerosols from cigarettes or other emerging products such as electronic nicotine delivery systems (ENDS), which are not representative of human exposure. In the present study, a whole aerosol (WA) system was used to expose lung epithelial cultures (2D and 3D) to determine the potential of six Vuse Alto ENDS products that varied in nicotine content (1.8%, 2.4%, and 5%) and flavors (Golden Tobacco, Rich Tobacco, Menthol, and Mixed Berry), along with a marketed ENDS and a marked cigarette comparator to induce cytotoxicity and oxidative stress. The WA from the Vuse Alto ENDS products was not cytotoxic in the NRU and MTT assays, nor did it activate the Nrf2 reporter gene, a marker of oxidative stress. In summary, Vuse Alto ENDS products did not induce cytotoxic or oxidative stress responses in the in vitro models. The WA exposures used in the 3D in vitro models described herein may be better suited than 2D models for the determination of cytotoxicity and other in vitro functional endpoints and represent alternative models for regulatory evaluation of tobacco products.

## 1. Introduction

Cigarette smoking is a preventable cause of many serious diseases and death. Inhalation of toxicants produced in the process of tobacco combustion is recognized as the driver of smoking-related diseases, such as cardiovascular diseases, chronic obstructive pulmonary disease (COPD), and lung cancer through perturbations in numerous biological pathways. For example, cellular and molecular mechanisms, including cell death, oxidative stress, and inflammation have been demonstrated to be contributing factors in individual cases of smoking-related disease [1]. Cigarette smoke is a dynamic aerosol of several thousand chemicals and toxicants which partition into a gas vapor phase (GVP) and a particulate phase; the particulate phase is known as total particulate matter (TPM). Complete quitting of all tobacco products including cigarette smoking is recognized as the best cessation approach [2] The harmful effects from smoking may be reduced by switching smokers who cannot or are unwilling to quit to alternative tobacco products which minimize exposure to the combustion-related toxicants [3].

Currently, all tobacco and nicotine products are regulated by the U.S. FDA. A number of toxicants present in cigarette smoke have been identified as harmful and potentially harmful constituents (HPHCs) by the US FDA [4]. In vitro non-clinical studies are among the various toxicological and mechanistic evaluations recommended for gaining approval for marketing new tobacco and nicotine products [5]. Since human smoking is difficult to replicate in laboratory experiments, fractions of cigarette smoke (GVP and TPM) or chemical evaluation of component HPHCs alone or in combination have been used in a variety of cell culture systems to assess the effects of tobacco products [6,7]. In vivo inhalation studies also have been used to evaluate the toxicological effects of exposure to cigarette smoke [8]. While the wealth of knowledge accumulated through these studies has substantially contributed to our understanding of the harmful effects of cigarette smoking, several technical and ethical limitations of these conventional test systems are also recognized [9].

The lungs are one of the main target organs impacted by cigarette smoking, and the human lung is composed of many types of well-differentiated cells which differ spatiotemporally in their localization and perform distinct functions. In contrast, cell lines and primary cells cultured in monolayers are submerged, not fully differentiated, and lack the functional features of their tissue of origin. Consequently air–liquid interface (ALI) cultures of lung cells that replicate several histological and functional features have been developed [10,11,12]. For example, ALI cultures of primary bronchial epithelial cells have been developed for the evaluation of relative cytotoxicity, ion channel functions, oxidative stress, inflammation responses, and gene expression changes under acute and repeat dose treatments of the different constituent phases of cigarette smoke and aerosol from electronic nicotine delivery systems (ENDS) [13,14,15,16,17].

Other limitations of conventional in vitro test systems include a lack of sensitivity to the composition of cigarette smoke aerosol changes with time, a process termed aging. Thus, cigarette smoke is best assessed as “freshly” as possible or as it is generated [18]. Further, both TPM and GVP contain HPHCs that are known to contribute to toxic effects of cigarette smoke, and testing one type of sample alone is considered only a partial representation of cigarette smoke toxicity [19]. Additionally, cell cultures submerged in liquid often do not provide accurate dosimetry, which is an important component of risk assessment [18,19]. Various programmable smoking robots such as those manufactured by Vitrocell were developed to deliver freshly generated whole aerosol (WA) from other battery-operated nicotine devices such as HTP and ENDS as well as whole smoke from a combustible cigarette to cells cultured under ALI. Currently, 3D cultures which are derived from human primary cells that mimic several histological and functional attributes of lung cells are available from different commercial sources (MatTek and Epithelix). Several investigators have used these 3D cell cultures to assess the effects of exposure to cigarettes and other tobacco products [20,21,22,23].

ENDS are non-combustible inhalable nicotine-containing products that are generally viewed as potentially less harmful than cigarettes [24,25] and have recently gained a significant market presence. ENDS encompass several types of electronic devices that may be paired with numerous flavored e-liquids. The long-term health effects of ENDS use are an active area of research. Vuse ENDS consist of several generations of ENDS products marketed by R.J. Reynolds Vapor Company, and three Vuse products with the tobacco flavor only are currently authorized for marketing through the PMTA pathway [5,26]. The toxicological, including genotoxic, effects of Vuse Alto ENDS products were determined in a battery of tests using well established regulatory recommendations and guidelines, and those findings are under consideration for publication elsewhere. In this manuscript, we have further evaluated WA from Vuse Alto products differing in nicotine strength and flavor relative to cytotoxic and oxidative stress from exposure to WA from a comparator cigarette smoke and a market comparator ENDS product. The cytotoxicity of Vuse Alto ENDS products was evaluated by three different in vitro assays that indicate different states of cell death in monolayer (2D) cultures and 3D EpiAirway tissues. Additionally, the potential of Vuse Alto ENDS products to induce oxidative stress was assessed in EpiAirway tissues using a novel Nrf2 luciferase reporter gene activation assay.

## 2. Materials and Methods

### 2.1. Test Products

This study evaluated potential cytotoxicity and oxidative stress from WA exposure to several Vuse Alto ENDS products that differed in nicotine concentration and flavor. Vuse Alto ENDS products are pod/mod-type ENDS products marketed by R.J. Reynolds Vapor Company and consist of closed e-liquid cartridges (also referred to as pods) that work in combination with a non-adjustable power unit with a rechargeable battery (typical capacity 370 mAh). The Vuse Alto pods are non-refillable and contain approximately 1.8 mL of e-liquids containing VG/PG, water, varying flavor ingredients, and salt-based nicotine contents of 1.8%, 2.4%, and 5.0% by weight. The following four flavors of Vuse Alto test products at 5% nicotine concentration were tested: Golden Tobacco, Rich Tobacco, Menthol, and Mixed Berry. In addition, the Golden Tobacco-flavored Vuse Alto product at 1.8% and 2.4% nicotine concentrations was also tested.

Commercially available market comparator ENDS (JUUL Mint 5%) and a combustible cigarette comparator (Marlboro Gold) were also assessed. The comparator products were market leading products in their respective categories at the time of the study [27,28].

### 2.2. Whole Aerosol Generation

Whole aerosol from Vuse Alto test products and the comparator products was generated using a Vitrocell^®^ VC10^®^ smoking robot (Vitrocell^®^ Systems GmbH, Waldkirch, Germany), which was connected to either a Vitrocell Mammalian 48 exposure module (H292 cells) (Vitrocell^®^ Systems GmbH, Waldkirch, Germany) or Vitrocell 12/4 modules (EpiAirway™) (Vitrocell^®^ Systems GmbH, Waldkirch, Germany) at ALI, as described previously [21,29,30,31]. A visible representation of the exposure set up has been detailed further previously for both H292 cells and EpiAirway™ [32,33,34]. The smoke/aerosol was exhausted from the aerosol generator then serially diluted to achieve different doses of exposure, with undiluted (0 L/min) or 0.5 L/min being the most concentrated and highest dose of exposure for ENDS or cigarette, respectively. Lower doses were achieved by higher flow rates resulting in dilution of WA. For each ENDS test product: incubator controls (negative), ALI controls (vehicle for WA/WS), positive controls, and whole smoke/aerosol exposures were tested with six (H292) or three (EpiAirway™) replicates. Concentrations of whole smoke/aerosol exposed to the tissues were expressed in terms of air flow in litres per minute (L/min) or nicotine equivalent units as described below. The vacuum rate was fixed at 5 millilitres per minute (mL/min). Exposure conditions for EpiAirway™ tissues were 4 to 0 (undiluted) L/min of diluting air for the ENDS and 10 to 0.5 L/min (EpiAirway™ tissues) and 8 to 0.5 L/min (H292) for the cigarette comparator.

Fresh whole smoke from the cigarette comparator was generated under the Health Canada Intense regimen (55 mL puff volume, 30 s puff interval, 2 s puff duration, with 100% vent blocking) [35]. Additional parameters included a puff exhaust duration of 8 s and a bell-shaped puff profile. Monolayer cells of H292 were exposed to cigarette smoke from 8 to 0.5 L/min dilutions of airflow for 24 min for the NRU assay. The exposure for EpiAirway™ cultures for the MTT assay was 68 min and 24 min for the LDH and Nrf2 luciferase reporter assays.

Aerosol from the Vuse Alto test products and the market comparator ENDS product was generated per a modified ISO20768:2018 [36]. The puffing parameters were puff volume, 55 mL; puff duration, 3 s; puff frequency, 30 s; puff profile, square wave; puff exhaust duration, 8 s; 180 puffs per ENDS cartridge. For H292 monolayer cultures, a total of 360 puffs of ENDS aerosol was generated from three pods, flow rates ranged from 4–0 L/min of diluted airflow, and the exposure time was 180 min. While the flow rates for EpiAirway™ cultures remained similar, ENDS aerosol was generated from 240 puffs and exposure time was 120 min. Because 60 s pauses were taken for clearing puffs for every 10 puffs, total exposure duration was longer than the actual exposure time to ENDS aerosols.

### 2.3. Photometers

Photometers were purchased from Vitrocell^®^ Systems, GmBH. For combustible cigarettes and ENDS, seven photometers for each product type, were harmonized to whole smoke using the regimens described in the WA generation section and a diluting airflow of 0.25 L/min so that the voltage for each photometer was approximately 4.0 v. Harmonization was performed as previously described [37]. Each photometer was connected to the VC Photometer Control Box, which was then connected to a computer. Photometers were used to confirm real time delivery of the aerosol/smoke to the module.

### 2.4. Chemical Analyses

#### 2.4.1. Nicotine Determination

Following the completion of exposures, nicotine content was determined from the whole smoke/aerosol-conditioned medium in each well of a dosimetry module, as described previously [31] at Broughton Nicotine Services (Barnoldswick, UK), using an internally validated method. Samples were processed on a Thermo Endura LC-MS/MS with a Dionex Ultimate 3000 low pressure quaternary analytical HPLC system (Waltham, MA, USA) fitted with a Waters XBridge BEH Shiled RD18 (2.5 µm) 3.0 × 500 mm analytical column (Milford, MA, USA). The limit of detection and quantification was 0.08 µg/mL with a range up to 50 µg/mL. The test products differ in their overall chemistry profiles; however, it has been published that nicotine concentrations are a key variable for smokers switching. The nicotine content of the WA (measured in the dosimetry modules) at each flow rate was used to compare the cultures across the Vuse Alto ENDS test products and the market comparators [38,39]. Thus, the exposures are presented in terms of μg of nicotine equivalent units [13,14,16]. Nicotine equivalents were calculated by multiplying the volume of the dosimetry well in the exposure module by the chemical determination of nicotine following the completion of the experiment.

#### 2.4.2. Carbonyl Determination

The whole smoke/aerosol-conditioned phosphate buffered saline from the cigarette comparator, Vuse Alto ENDS test products, and the comparator ENDS test products was used for the determination of carbonyl compounds. The trapped carbonyls in the condition medium were derivatized with 2,4-dinitrophenylhydrazine (DNPH). Samples were performed using a Thermo Endura LC-MA/MS with a Dionex Ultimate 3000 low pressure quaternary analytical HPLC system (Waltham, MA, USA) fitted with a Waters Acquity BEH Shield C18 (1.7 µm) 2.1 × 500 mm analytical column (Milford, MA, USA). The analytical methodology was derived from the method described previously [40]. The nominal quantifiable range for each carbonyl compound was 0.01 to 3.9 µg/mL, with a limit of detection at 0.003 µg/mL. The carbonyls that were quantified were acetaldehyde, acrolein, crotonaldehyde, and formaldehyde.

### 2.5. Transepithelial Electrical Resistance (TEER)

Prior to the TEER assessments, tissues were washed to remove any accumulated mucus on the apical surface by rinsing with Dulbecco’s phosphate-buffered saline (D-PBS). TEER was measured with an EVOM2 epithelial Volt/Ohm meter per the manufacturer’s instructions. EpiAirway™ tissues were accepted for use in the study if the TEER value was greater than 300 Ohm*cm^2^ (manufacturer’s acceptance criteria).

### 2.6. Determination of Cytotoxicity

Three different assays were utilized to evaluate the cytotoxicity of Vuse Alto test products using H292 monolayer cells and EpiAirway™ 3D cultures (MatTek Corporation, Ashland, MA, USA). While Triton-X100 was used as a positive control in all the three cytotoxicity assays, heptyl butyrate (200 mg/mL in olive oil) and formaldehyde (14 mg/mL in media) were also included as additional positive controls for assays using EpiAirway™ cultures [21]. Negative controls include ALI cells exposed to flowing air and incubator and vehicle controls.

#### 2.6.1. NRU Assay

Monolayer cultures of NCI-H292 cells, supplied by the European Collection of Authenticated Cell Cultures (ECACC), were used to assess cytotoxic responses when exposed to WA from Vuse Alto ENDS products. Briefly, the cells were exposed to cigarette smoke for 24 min or ENDS products for 180 min at ALI, and cytotoxicity was assessed using the NRU method after 24 h. A calculation of cell viability expressed as neutral red (dye) uptake was made for each condition using the mean neutral red absorbance of the replicate Transwells™, corrected by the mean blank Transwell™ value. Although no regulatory guidelines exist for this assay with WA exposures, guidance was taken and run in general accordance with a protocol previously described by ICCVAM and a published whole aerosol NRU study [29,41].

The main criteria for determining a cytotoxic response in a replicate assay included (1) a concentration-related decrease in neutral red uptake over the dose range tested with (2) at least a 50% reduction in neutral red absorbance compared to the ALI control. A test product which gave a cytotoxic response across at least two of the main experiments (i.e., at least a 50% reduction in neutral red absorbance compared to the ALI control is observed) was considered overall as cytotoxic.

#### 2.6.2. MTT Assay

EpiAirway™ tissues from MatTek corporation (Ashland, MA, USA) were maintained in 12 mm Transwells™ at ALI. Exposure time for the ENDS products was approximately 120 min for ENDS and 68 min for cigarette test products. After exposure to smoke from cigarettes or whole aerosol from ENDS, tissues were returned to the incubator and maintained for 24 h post exposure prior to assessment using the MTT (3-(4,5 dimethylthiazol-2-yl)-2,5-diphenyltetrazolium bromide) assay to determine cytotoxicity as described previously [21]. Cytotoxicity was defined as a greater than 50% decrease in relative survival compared to the concurrent ALI control.

### 2.7. EpiAirway™ Nrf2 Tissue Model

The Nuclear Factor Erythroid 2-Related Factor 2 (Nrf2) gene is a transcription factor that responds to oxidative stress and promotes transcription of genes that protect cells from oxidative stress [42]. Briefly, EpiAirway™ tissues are stably transfected with a lentiviral vector consisting of the antioxidant responsive element of the Nrf2 gene fused with the luciferase gene as a bioreporter. The EpiAirway™ Nrf2 tissue model was developed by MatTek corporation and was described previously [21]. Additional controls were included for Nrf2 tissue model: Cobalt (II) Chloride (0.05% in water) and t-Butyl Hydroquinone (250 and 500 µM in 1:1 DMSO:PBS), vehicle controls for each, and incubator controls as negative controls.

#### LDH Assay

Briefly, luciferase-transfected EpiAirway™ tissues are assessed for cytotoxicity using the lactate dehydrogenase (LDH) assay following exposure to whole aerosol/smoke from ENDS test articles and one cigarette test article utilizing a modified ISO20768:2018 and Health Canada Intense (HCI) smoking regimen [35], respectively. EpiAirway™ tissues were maintained in 12 mm Transwells™ at the ALI. The vacuum rate was fixed at 20 millilitres per minute (mL/min). The exposure conditions were 3 to 0 (undiluted) L/min of diluting air for the ENDS and 6 to 0.5 L/min for the comparator cigarette.

Culture media from each control and test samples were collected after the 18 h post-exposure period and were analyzed using the LDH Cytotoxicity Detection Kit (Clontech Cat# 630117/Takara Cat# MK401) following the manufacturer’s instructions.

### 2.8. Determination of Oxidative Stress in 3D EpiAirway™ Cultures

After WA exposure, the EpiAirway™ tissues were returned to the incubator and maintained for 18 h post exposure prior to determining the activation of the Nrf2 luciferase (Nrf2) reporter, as described previously [21]. A time course (6, 18, 24, 48 h) of post exposure was assessed previously using both ENDS and combustible cigarettes, and the 18 h post exposure time was determined to have the highest induction of oxidative stress in this model [21].

Luciferase activity was measured using the ONE Glo^TM^ Luciferase Report Assay System (Promega, UK) per the manufacturer’s instructions. Briefly, following post-exposure incubation, tissues were gently rinsed with Dulbecco’s phosphate-buffered saline to remove mucus from the apical surface. The ONE Glo^TM^ Reagent was added onto the apical surface of each tissue, and cultures were incubated at room temperature for at least 15 min, protected from light to allow for complete cell lysis. Following lysis, an aliquot of the lysate was transferred to a white-walled 96-well microplate, and luminescence (luciferase activity) was recorded using a SpectraMax L plate reader. Activation of the Nrf2 luciferase reporter gene is expressed in relative luminescence units (RLUs).

#### Data Analysis and Statistical Methods

Oxidative Stress: For each experiment and product, linear interpolation was used to determine the lowest nicotine concentration at which a 2-fold increase in maximum fold change was achieved (2-fold concentration). The log-transformed 2-fold concentrations determined for the test articles were compared using *t*-tests, with a *p* < 0.05 being considered significant. Comparisons were made between the combustible cigarette and each ENDS test article using the SAS^®^ program (Cary, NC, USA).

NRU and MTT endpoints: For each test article in which a 50% reduction in mean survival relative to the air control was achieved, IC_50_ was a sigmoidal model with the top parameter fixed at 100 and bottom parameter fixed at 0. Comparison of IC_50_ values between the market combustible and each ENDS test article was performed using *t*-tests of mean log-transformed IC_50_ values, with a *p* < 0.05 being considered statistically significant using the SAS^®^ program (Cary, NC, USA).

## 3. Results

### 3.1. Whole Aerosol Studies

A VC10 Whole Aerosol system was used to evaluate the effects of freshly generated aerosol from the Vuse Alto ENDS products for potential cytotoxic and oxidative stress effects in human monolayer and 3D cell cultures. The WA studies were conducted with Vuse Alto Golden Tobacco at 5%, 2.4%, and 1.8% nicotine concentrations and Vuse Alto Rich Tobacco, Menthol, and Mixed Berry at 5% nicotine concentrations, along with the two comparators.

Media from the dosimetry modules from undiluted WA exposures were analyzed for nicotine and four carbonyl compounds to determine the dose range of cell exposure (Table 1). The whole smoke (WS) from the cigarette comparator contained nicotine and the four carbonyl compounds. Whereas the nicotine content was lowest (14.37 ± 2 μg/mL), the levels of carbonyls were highest in the aerosol-conditioned medium from cigarettes. In contrast, nicotine levels in samples exposed to Vuse Alto ENDS products were significantly higher than from cigarettes and generally aligned with the nicotine concentrations of the Vuse Alto ENDS test products. The conditioned medium from Vuse Golden Tobacco products with 1.8% and 2.4% nicotine concentrations contained 343 ± 27 and 458 ± 19 μg/mL, respectively. The four Vuse Alto ENDS products with 5% nicotine concentration (Golden Tobacco, Rich Tobacco, Menthol, and Mixed Berry flavors) contained 878 ± 160 to 1086 ± 74 μg/mL of nicotine. The conditioned medium from the exposure to WA from the marketed ENDS comparator contained a nicotine content of 108.8 ± 21 μg/mL, which was higher than for the cigarette comparator but lower than any of the Vuse ENDS products. As shown in Table 1, the levels of formaldehyde were three times lower in samples exposed to the Vuse Alto ENDS products or the marketed comparator ENDS product compared with cigarettes. The levels of the other three carbonyls were either below the levels of detection (LOD) or quantification (LOQ) in the ENDS products. In three instances where carbonyl compounds were at detectable levels in the WA of Vuse Alto ENDS products (two for acetaldehyde and one for acrolein), their levels were markedly lower relative to comparator WS from cigarettes.

Table 1 Chemical analyses of whole aerosol from Vuse Alto test products and the comparator products; nicotine, formaldehyde, acetaldehyde, acrolein, and crotanaldehyde levels analyzed in the undiluted whole aerosol (ENDS) or 0.5 L/min (combustible)-diluted the test products were determined as described in Materials and Methods at the end of the experiments in the dosimetry trap. The limit of detection (LOD) level for all analytes was 0.003 µg/mL. Mean and standard deviation values from mean of three experiments are presented.

#### 3.1.1. NRU Assay

**Figure 1:**
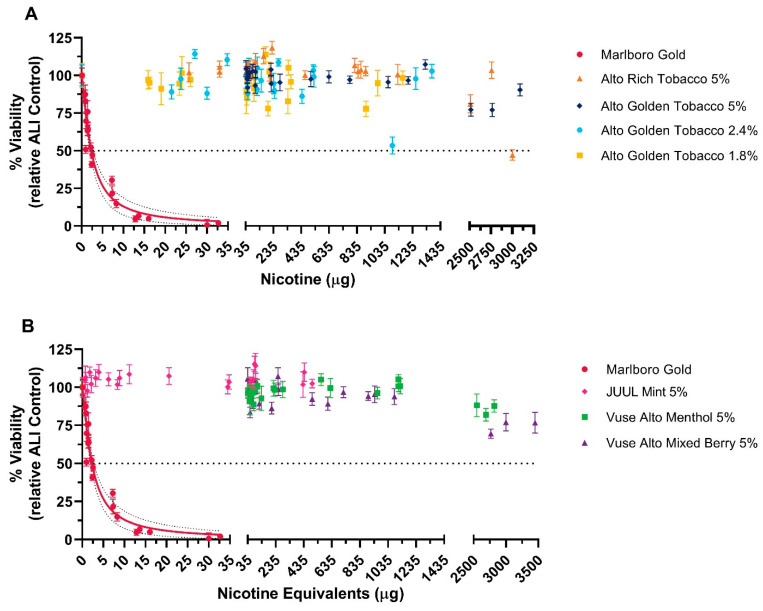
Determination of the cytotoxicity of Vuse Alto ENDS products and the market comparators, NRU assay. Monolayers of H292 cells were exposed to whole aerosol from the test articles, and cytotoxicity was determined using the neutral red uptake method (triplicate experiments, six replicates per airflow). Results from Alto Rich Tobacco 5% and Alto Golden Tobacco 5%, 2.4%, and 1.8% are shown in (**A**), whereas JUUL Mint 5%, Alto Mixed Berry 5%, and Alto Menthol 5% are shown in (**B**); data from the Marlboro Gold experiments are shown in both (**A**,**B**). A dotted line represents 50% cell death, designated as a threshold for cytotoxicity. A viability response curve with a 95% CI (dotted line) was plotted for any test article that induced a 50% reduction in cell viability in at least two experiments.

Monolayers of H292 cells were exposed to WA from the Vuse Alto ENDS and the comparator products, and cytotoxicity was determined by the NRU method after 24 h (Figure 1). Visual examination of cultures immediately following exposure to WS from cigarette smoke caused noticeable toxicity at lower flow rates (≤2.0 L/min), as evidenced by the presence of floating and rounded cells. A decrease in the toxicity of cigarette smoke was observed incrementally from flow rates of 8 L/min to 2 L/min immediately after exposure. Following 24 h of exposure to cigarette smoke, cytotoxic effects were evident with the presence of cells having poor morphology and/or floating cells. Cell survival increased with increasing flow rates (increasing dilution of cigarette smoke or ENDS WA) and ≥80% live cells at an 8 L/min flow rate. It was determined that exposure to WS of cigarette smoke resulted in cell death with a IC_50_ value of 2.28 ± 0.18 μg of nicotine equivalent units. These toxic effects led to the conclusion that exposure to cigarette whole smoke was cytotoxic. Similarly, 1% Triton-X-100, a known cytotoxin, caused 100% cell death following exposure, which confirmed appropriate sensitivity of the assay.

Examination of cells immediately following exposure to Vuse Alto revealed the presence of rounded cells with undiluted aerosols. However, with airflow increased to ≥ 1.0 L/min, the monolayers were rarely disturbed, and cell morphology was unaltered. While the Vuse Alto products caused some morphological alterations at <1.0 L/min, cell survival with undiluted aerosol was >50% with WA exposure to all ENDS test products. Exposure to undiluted and diluted aerosols across the Vuse Alto ENDS products was not cytotoxic. The aerosol from the comparator ENDS product also produced similar effects as the Vuse ENDS products. Relative to the calculated IC_50_ value for the combustible comparator, the maximum delivered doses of the Vuse Alto test products ranged from 1160–3450 μg nicotine equivalent units. Within this range, none of the Vuse Alto test products generated adequate cytotoxicity to calculate an IC_50_ value.

#### 3.1.2. MTT Assay

**Figure 2:**
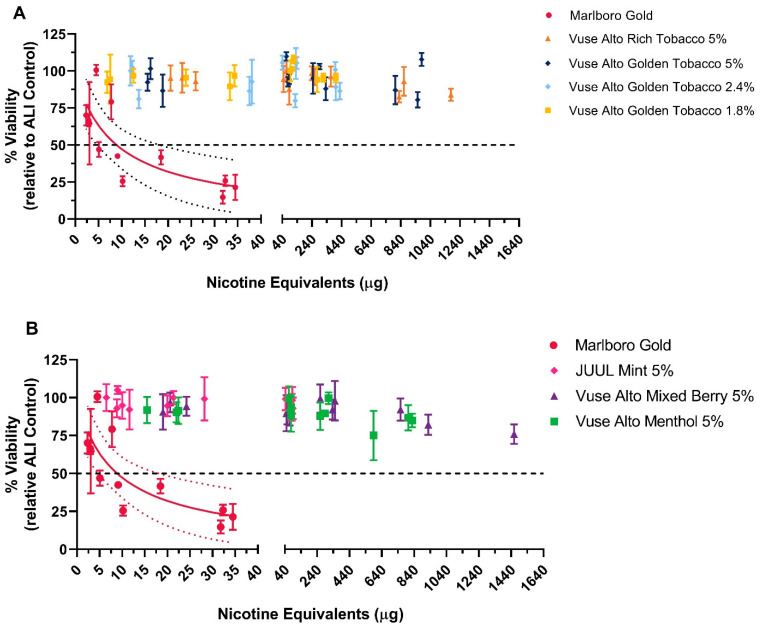
Determination of the cytotoxicity of Vuse Alto ENDS products and the market comparators, MTT assay. EpiAirway™ tissues were exposed to whole aerosol from the test, and cytotoxicity was determined through MTT assay (triplicate experiments, three replicates per airflow). Results from Alto Rich Tobacco 5% and Alto Golden Tobacco 5%, 2.4%, and 1.8% are shown in (**A**), whereas JUUL Mint 5%, Alto Mixed Berry 5%, and Alto Menthol 5% are shown in (**B**); data from the Marlboro Gold experiments are shown in both (**A**,**B**). A horizontal dotted line represents 50% cell death, designated as a threshold for cytotoxicity. A viability response curve with 95% CI (dotted line) was plotted for any test article that induced a 50% reduction in cell viability in at least two experiments.

The EpiAirway™ tissues were exposed to varying concentrations of WA from the six Vuse Alto ENDS products and market comparator products for cigarette and ENDS. Visual observation immediately following exposure of the tissues to the WS from the comparator cigarette showed dryness of the tissue surface at lower dilutions and in the presence of 0.5 L/min cigarette smoke. Consistent decreases in MTT greater than 50% were observed at airflows of 4 L/min and 1 L/min for the comparator cigarette product with an IC_50_ average of 8.89 ± 2.26 μg nicotine equivalent units (Figure 2).

Immediate visual observation of EpiAirway™ tissues exposed to aerosol from Vuse Alto ENDS products or the comparator ENDS product revealed that the tissues were generally healthy at all concentrations of the aerosol tested. In contrast to the comparator cigarette products, Vuse Alto ENDS products and the comparator ENDS product did not cause a >50% reduction in MTT at any of the concentrations (air dilutions) tested and were not considered to be cytotoxic.

#### 3.1.3. LDH Release Assay and Oxidative Stress

**Figure 3:**
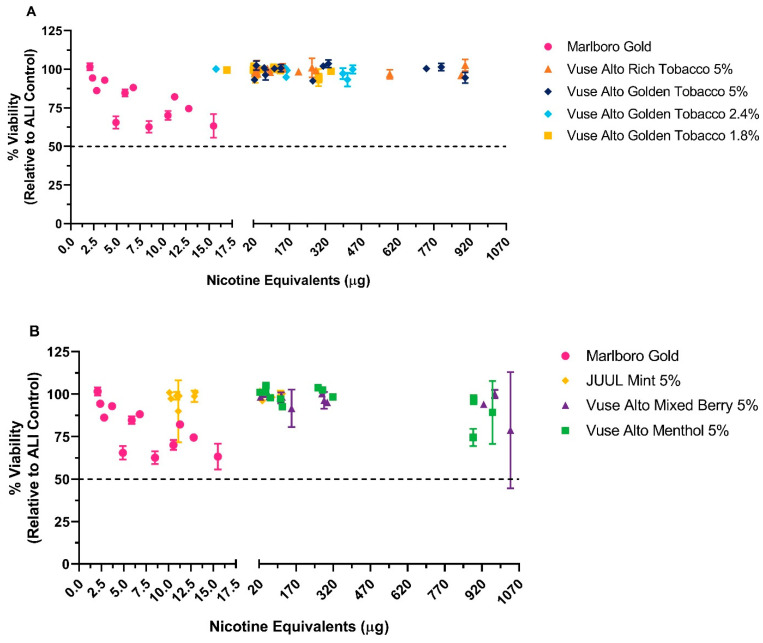
LDH release (cytotoxicity) was measured from whole aerosol exposures to Vuse Alto ENDS products and the market comparator products. EpiAirway™ Nrf2 tissues were exposed to whole aerosol or whole smoke as described in Materials and Methods. Results from Alto Rich Tobacco 5% and Alto Golden Tobacco 5%, 2.4%, and 1.8% are shown in (**A**), whereas JUUL Mint 5%, Alto Mixed Berry 5%, and Alto Menthol 5% are shown in (**B**); data from the Marlboro Gold experiments are shown in both (**A**,**B**). A horizontal dotted line represents 50% cell death. Whole smoke/aerosol was expressed in nicotine equivalent units. LDH release was measured in the basolateral media following an 18 h post-exposure recovery prior to the evaluation of Nrf2 expression (Figure 4). Cell survival (mean and standard deviation) was determined from three independent experiments with three replicate tissues per airflow.

The LDH release assay was run concurrently with the Nrf2 oxidative stress assay to ensure that the tissues were not exposed to cytotoxic doses (i.e., >50% cell death) of WA from the test products. The detection of Nrf2 was based on the production of a Nrf2 luciferase-linked transfected plasmid. Cell viability and oxidative stress were assessed in the same EpiAirway™ tissue preparations exposed to the test products. Eighteen hours following exposures, dose-dependent changes in cell viability as measured by LDH release were observed only for the market cigarette comparator (Figure 3). Exposures/dosing were measured by the nicotine content in dosimetric modules, and real time exposure was confirmed by photometers. A dose-dependent decrease in cell viability (101.6 ± 2.4% to 63.4 ± 7.6%) was observed as the WA dose increased from 2.1 to 15.5 μg of nicotine equivalent units. In contrast, exposure to Vuse Alto ENDS and ENDS comparator products resulted in a minimal loss of cell viability, and no dose-dependent changes were detected. These data show that exposures to WA from cigarette comparators at the highest dose remained below the cytotoxicity threshold of 50% loss of cell viability.

**Figure 4:**
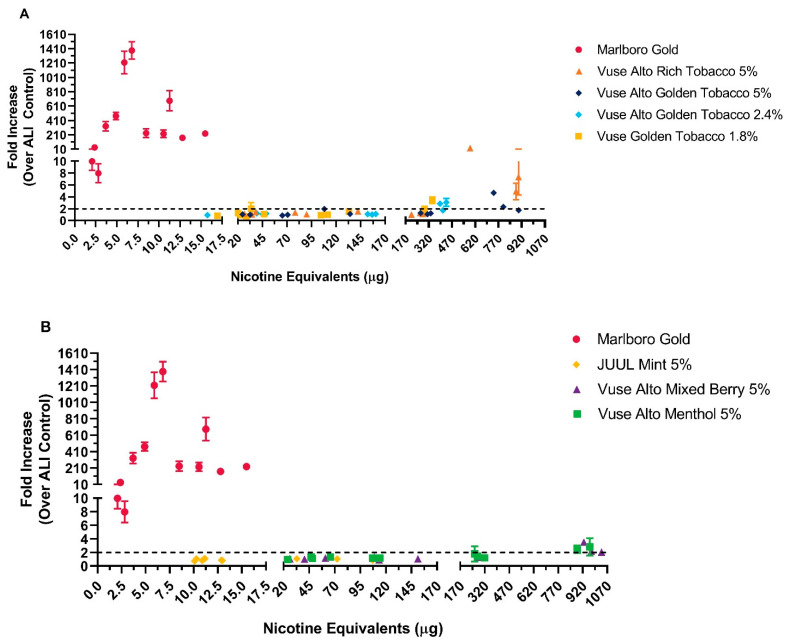
Activation of the Nrf2 reporter gene by Vuse Alto ENDS products and the market comparator products. EpiAirway™ Nrf2 tissues were exposed to whole aerosol as described in Materials and Methods. Results from Alto Rich Tobacco 5% and Alto Golden Tobacco 5%, 2.4%, and 1.8% are shown in (**A**), whereas JUUL Mint 5%, Alto Mixed Berry 5%, and Alto Menthol 5% are shown in (**B**); data from the Marlboro Gold experiments are shown in both (**A**,**B**). Whole smoke/aerosol dosing is expressed as nicotine equivalent units. Induction of oxidative stress is measured by relative luminescence of the activated Nrf2 luciferase reporter gene. Mean and standard deviation from three independent experiments were calculated with three replicates per airflow. The relative fluorescence from >2-fold increases over the ALI controls (dotted line) was considered to induce oxidative stress.

A concentration-dependent increase in the Nrf2 reporter gene in samples exposed to WA from the cigarette comparator was evident from profound increase in the luciferase activity, indicating induced oxidative stress (Figure 4). The reporter gene activity increased ranged from from 10 ± 1.5-fold to 1385 ± 121-fold as the WA concentration increased from 2.1 to 15.5 μg of nicotine equivalent units. Higher doses of WA, however, resulted in a decrease in the Nrf2 reporter activity; at the highest dose of 15.5 μg of nicotine equivalent units of whole aerosol, the reporter activity showed a 229.1 ± 41.3-fold increase, indicating, although decreased, still notably higher oxidative stress levels than those observed in any of the test samples. The WA from the ENDS market comparator up to 114 μg of nicotine equivalents did not induce more than a 2-fold increase in Nrf2 reporter gene activity (i.e., threshold for inducing oxidative stress).

In general, the Nrf2 activity in EpiAirway™ tissues exposed to WA from the Vuse Alto ENDS products was at background levels or significantly lower than with the WS from the cigarette comparator; the only increases in the reporter gene activity, over the background, were at the highest doses of whole aerosol from some Vuse Alto products (Figure 4). For example, increased Nrf2 reporter gene activity was observed in Vuse Alto Golden Tobacco 2.4% (3.1 ± 0.7 fold increase at 433 μg/mL of μg of nicotine equivalent units), Rich Tobacco 5% (7.4 ± 3.1 fold increase at 900 μg/mL of μg of nicotine equivalent units), Menthol 5% (2.8 ± 1.3 fold increase at 963 μg of nicotine equivalent units), and Mixed Berry 5% (2.2 ± 0.4 fold increase at 1035 μg of nicotine equivalent units). All other test conditions with the whole aerosol from the six Vuse Alto ENDS products did not induce Nrf2 reporter gene activity. Taken together, whole aerosol from some Vuse Alto ENDS products in this study induced oxidative stress in the EpiAirway™ model at only the highest tested dose.

## 4. Discussion

A goal of this research was to implement known WS/WA methods as alternative, non-animal test methods for regulatory assessment of emerging non-combustible tobacco and nicotine products. In this manuscript, we report the evaluation of cytotoxicity and oxidative stress from exposure to several Vuse Alto ENDS products using a WA exposure system. Key findings from this study are (1) the Vuse Alto ENDS products which varied in nicotine concentration and flavors did not induce cytotoxicity as determined by three different assays; (2) relative to cigarettes, the Vuse Alto ENDS products did not elicit oxidative stress responses in most instances, while substantially reduced responses were detectable only at the highest doses.

Evaluation of toxicological effects is mandatory for seeking marketing approval for tobacco products in the U.S. [5]. The U.S. FDA guidance on premarket tobacco applications for ENDS products recommends inclusion of non-clinical studies, among others, for the assessment of health risks of tobacco products [5]. Previous reports indicate the cytotoxic and genotoxic effects of exposure to ENDS products, in general, are significantly lower compared to cigarettes [21,23,43], while some investigators have reported the toxicological effects of ENDS (e.g., [44]. Regardless, it is necessary to demonstrate that candidate tobacco products proposed for marketing do not increase health risks; hence, a thorough evaluation of new tobacco products is required.

The six Vuse Alto ENDS products tested in this study ranged in nicotine concentration from 1.8% to 5% and comprised Golden Tobacco, Rich Tobacco, Menthol, and Mixed Berry flavors. Compared to the market comparator cigarette and ENDS products, the WA from all Vuse Alto ENDS products tested here contained higher levels of nicotine. There is a dose-dependent increase in the nicotine yields of Vuse Alto ENDS products’ WA. The whole smoke from cigarettes, consistent with the established literature [45,46], contained formaldehyde, acetaldehyde, acrolein, and crotanaldehyde, to which are attributed many of the in vitro toxicological effects of cigarette smoke [1,47,48]. Independent of nicotine yield, the WA from Vuse Alto ENDS contained these four carbonyl compounds at LOD and LOQ levels, or at significantly lower levels than what is found in WA cigarette aerosol when detectable. Others have reported lower levels of carbonyls for Vuse Alto ENDS when compared to a combustible cigarette; however, only the 5% rich tobacco was used for these assessments [49]. Formaldehyde is an established HPHC in cigarette smoke and is identified as a respiratory irritant and carcinogen [4]. Several variables, including device type and operating conditions (e.g., temperature) have been known to influence the levels of formaldehyde in the ENDS emissions [50,51]. For example, formaldehyde levels were found to be lower in JUUL aerosol (comparator ENDS product) relative to other e-cigarette types and cigarette smoke [50,52].

Here we report that the exposure to formaldehyde is lower in the Vuse Alto products than from the cigarette comparator. Compared to JUUL, the exposure to formaldehyde, although slightly lower, was comparable for the Vuse Alto ENDS. Thus, our results are consistent with the existing scientific literature.

While analyses of WA from the Vuse products were limited to these four carbonyl compounds, given that Vuse ENDS do not combust, reductions in other HPHCs compared with cigarettes are also likely. It is relevant to note that Margham et al., reported that aerosol from e-cigarettes is compositionally less complex than cigarette smoke and contains significantly lower levels of toxicants [53,54]. The significant reductions in these four carbonyl HPHCs in the Vuse Alto ENDS is expected to be reflected in marked reductions in some of the adverse responses, such as cytotoxicity and oxidative stress, associated with the exposure to cigarette smoke.

Cytotoxicity is one of the well-established adverse effects of exposure to cigarette smoke or its constituents, and several methods of evaluating cytotoxicity exist [6]. For example, Putnam et al. evaluated the cytotoxicity of TPM from cigarette smoke on monolayer CHO cells by eight different methods [55]. The NRU assay has been widely used and regulatory guidelines exist for this assay with the Chinese hamster ovary cell system. With an overarching goal of utilizing human cells for the assessment of tobacco products, we adapted the NRU assay with H292 cells [29]. For the EpiAirway, we previously developed an MTT assay for cytotoxicity [21], as it was used together with EpiAirway for developing in vitro models of inhalation toxicity [56]. Here, we evaluated the cytotoxicity of Vuse Alto ENDS products and comparators for a cigarette and ENDS by tests that assess cell viability and proliferation (NRU and MTT assays) and membrane integrity (LDH assay), which all measure cytotoxicity via different mechanisms. As expected, the NRU and MTT assay results consistently demonstrated cytotoxic effects of exposure to whole smoke from the comparator cigarette. Cigarette smoke, although inducing measurable cell death, was not cytotoxic per the criteria (>50% increase in toxicity) in LDH assays under the conditions of WA exposure. This finding is in line with the previous reports indicating that the LDH assay was less sensitive in longer exposure times for monolayer cultures treated with TPM preparations [55]. Importantly, the LDH assay was included in this study to ensure that the dose of WS from cigarettes was below the cytotoxic threshold and was concurrently run with oxidative stress assays.

Exposure to WA from all the Vuse Alto ENDS products for monolayer or EpiAirway cultures did not impact cell viability and was not cytotoxic. Although exposure to undiluted aerosol from the Vuse Alto ENDS products altered the morphology of some cells, it was not cytotoxic. The Vuse Alto ENDS products were not cytotoxic in any of the three assays in this study. Similar to our findings with the Vuse Alto products, other researchers have also not observed cytotoxicity with other ENDS products using WA exposure systems [20,23,29,43].

Numerous clinical and in vitro studies have demonstrated that cigarette smoke increases oxidative stress, which is an important driver of smoking-related diseases [1,57]. While several different methods exist for the determination of the burden of oxidative stress, here we used a method that incorporates WA-exposed 3D human airway tissues and a sensitive reporter gene assay. EpiAirway™ tissues transfected with the Nrf2 luciferase reporter responded robustly to exposure to cigarette whole smoke at sub-cytotoxic doses, indicating the induction of the oxidative stress response. Exposure to WA from the Vuse Alto ENDS products did not elicit oxidative stress responses, while substantially reduced responses were detectable only at the highest doses. These findings with Vuse Alto ENDS products are consistent with those from several other ENDS studies performed using different measures of oxidative stress [13,17,21,58].

It should be noted that there are reports indicating that ENDS induce oxidative stress (e.g., [59]). The product characteristics such as e-liquid composition, aerosol chemistry, flavorings, and operating conditions vary significantly and influence the toxicity profiles of e-cigarettes [60]; hence, e-cigarettes warrant a through toxicological evaluation. The Vuse Alto ENDS products evaluated in this study were neither cytotoxic nor inducers of oxidative stress relative to cigarettes in the WA exposure system at comparable or substantially higher nicotine equivalent doses. Other adverse effects (e.g., DNA damage, cell cycle arrest) were not evaluated in these experiments but have been examined in other regulatory assays (e.g., Ames, in vitro micronucleus) by the authors (data not shown, manuscript in preparation). However, evaluation of these effects in these models was not performed and is a limitation of this work; future experiments could measure these endpoints in these models. Others have begun to develop the micronucleus (MN, genotoxicity) using EpiAirway™ for aerosol exposures which would provide evidence for these potential effects [61].

While there are no currently established regulatory guidelines for the WA exposure studies, it is widely recognized that the WA exposure systems are preferred for in vitro regulatory purposes due to the ability to expose WS, which mimics human exposure [10,12,19]. However, optimization and standardization of WA aerosol exposure systems continues to evolve for in vitro toxicology testing of tobacco products, as is evident from this and other research [21,22,29,31,34,46,62]. Given the complex nature of cigarette whole smoke, one of the important advantages of the WA exposure systems over the more traditional test samples of fractionated particulate and gas vapor phases of smoke/vapor aerosols is the exposure to freshly generated constituents mimicking what consumers experience on acute exposure.

The ALI systems more closely represent in vivo conditions in humans than the standard cell cultures [63]. These models have been extensively utilized by government and other researchers in tobacco and nicotine product research. For example, gene expression changes and lung disease-related endpoints were evaluated by FDA scientists in ALI systems following exposure to cigarette smoke toxicants [64,65,66,67]; further, the ALI models have been combined with the WA aerosol exposure systems for cigarette smoke and formaldehyde [67,68]. Several other groups, including this one, utilized ALI systems with or without WA exposure systems for investigation of cytotoxicity, ion channel function, oxidative stress, and gene expression changes following exposure to cigarette smoke or e-cigarette preparations [13,14,16,20,23].

Among the strengths of the current study are the assessment of cytotoxicity by several methods in monolayer and 3D cultures with WA exposure systems, which are alternative models for animal testing. A limitation of the test systems is a lack of clear regulatory guidelines for tobacco product evaluations. However, a recent interlaboratory survey has identified variables with the in vitro exposure systems and also identified pockets of harmony among the testing laboratories [69]. Further, previous work from this group has led to standardization of methods for WA exposure and ALI cultures [21,31,34].

## 5. Limitations

The limitations of this work include the fact that whole smoke/aerosol exposures were only based a single acute exposure versus multiple/chronic post exposure times. This work did not evaluate the other pathways/downstream effects that are involved in cell death (e.g., caspase activation, cell cycle arrest) or oxidative stress (e.g., DNA damage, cytokine release). Since no regulatory guidelines exist for whole smoke/aerosol exposures of tobacco products, there is difficulty in comparing the results from different authors. Additionally, no experiments involved exposure to both cigarette whole smoke and ENDS whole aerosol, which could provide insight into the potential effects to dual users.

## 6. Conclusions

In conclusion, the aerosols of six Vuse Alto ENDS test products, relative to cigarette whole smoke, delivered higher amounts of nicotine at the end of the experiment but significantly lower amounts of acrolein, acetaldehyde, formaldehyde and crotanaldehyde; a component of these differences was the exposure times (≥2x than combustible cigarettes). Over these acute exposures, the Vuse Alto ENDS products that varied in nicotine concentration or flavors were not cytotoxic or inducers of oxidative stress under the test conditions.

## Figures and Tables

**Table 1:** Chemical analyses of whole aerosol/smoke from Vuse Alto ENDS products and comparator products.

Test Product	Nicotine (µg/mL)	Formaldehyde	Acetaldehyde	Acrolein	Crotonaldehyde
		(µg/mL)	(µg/mL)	(µg/mL)	(µg/mL)
Marlboro Gold	14.37 ± 2.25	1.52 ± 0.23	6.59 ± 0.65	0.56 ± 0.08	0.73 ± 0.06
Vuse Alto Golden Tobacco 5%	903.67 ± 73.71	0.51 ± 0.10	<LOQ	<LOQ	<LOQ
Vuse Alto Golden Tobacco 2.4%	458 ± 18.38	0.37 ± 0.04	<LOQ	<LOQ	<LOQ
Vuse Alto Golden Tobacco 1.8%	343 ± 26.88	0.4 ± 0.13	<LOQ	<LOQ	<LOQ
Vuse Alto Menthol 5%	1012.67 ± 40.55	0.28 ± 0.09	<LOQ	<LOQ	<LOQ
Vuse Alto Mixed Berry 5%	1086.67 ± 49.22	0.40 ± 0.08	0.325 ± 0.09	<LOQ	<LOQ
Vuse Alto Rich Tobacco 5%	878 ± 159.98	0.34 ± 0.065	0.26 ± 0.04	0.07 ± 0.01	<LOQ
JUUL Mint 5%	108.77 ± 20.39	0.5 ± 0.06	<LOQ	<LOQ	<LOQ

## Data Availability

The datasets presented in this article are not readily available because the data are part of an ongoing regulatory application. Requests to access the datasets should be directed to the corresponding author (Brian Keyser).
